# Prefrontal Contribution to Decision-Making under Free-Choice Conditions

**DOI:** 10.3389/fnins.2017.00431

**Published:** 2017-07-26

**Authors:** Shintaro Funahashi

**Affiliations:** Kokoro Research Center, Kyoto University Kyoto, Japan

**Keywords:** prefrontal cortex, decision-making, free-choice, choice-predictive activity, spontaneous fluctuation

## Abstract

Executive function is thought to be the coordinated operation of multiple neural processes and allows to accomplish a current goal flexibly. The most important function of the prefrontal cortex is the executive function. Among a variety of executive functions in which the prefrontal cortex participates, decision-making is one of the most important. Although the prefrontal contribution to decision-making has been examined using a variety of behavioral tasks, recent studies using fMRI have shown that the prefrontal cortex participates in decision-making under free-choice conditions. Since decision-making under free-choice conditions represents the very first stage for any kind of decision-making process, it is important that we understand its neural mechanism. Although few studies have examined this issue while a monkey performed a free-choice task, those studies showed that, when the monkey made a decision to subsequently choose one particular option, prefrontal neurons showing selectivity to that option exhibited transient activation just *before* presentation of the imperative cue. Further studies have suggested that this transient increase is caused by the irregular fluctuation of spontaneous firing just *before* cue presentation, which enhances the response to the cue and biases the strength of the neuron's selectivity to the option. In addition, this biasing effect was observed only in neurons that exhibited sustained delay-period activity, indicating that this biasing effect not only influences the animal's decision for an upcoming choice, but also is linked to working memory mechanisms in the prefrontal cortex.

## Introduction

Executive functions can be defined as the coordinated operation of various cognitive neural processes and allows to accomplish a current goal flexibly. Planning, judgment, decision-making, anticipation, and reasoning are examples of executive functions. To achieve proper judgment, correct decision-making, or timely action, a top-down control process is needed to control various neural operations in a coordinated and flexible manner. This top-down control process is called executive control. The prefrontal cortex is known to be an important brain area for executive control (Stuss and Benson, 1986), since it has been shown that human patients with damage to the prefrontal cortex exhibit poor judgment, planning, and decision-making (Stuss and Benson, 1986; Goldman-Rakic, 1987; Mesulam, 2000; Fuster, 2015). Notably, as Mesulam (2000) indicated, although massive damage to the prefrontal cortex produces no impairment in sensation, perception, and motor control, patients with prefrontal damage tend to reach closure prematurely, jump to conclusions on the basis of incomplete information, perseverate, and find it difficult to explore alternative solutions to the same problem. Since these deficits are not caused simply by a failure of perception, recognition, or memory, it has been thought that these deficits must be caused by a deficit of executive functions (Stuss and Benson, 1986; Mesulam, 2000). The prefrontal cortex has dense anatomical connections to posterior association cortices, limbic cortices, and subcortical structures (Petrides and Pandya, 1994; Fuster, 2015). Through these connections, the prefrontal cortex is able to monitor and control the operation of these brain areas, such as activating certain networks, inhibiting other networks, and integrating interactions among networks.

To understand the prefrontal contribution to executive functions, it is important to examine how executive control operates in the prefrontal cortex and what is the nature of the neuronal mechanism of executive control (Funahashi, 2001, 2017; Funahashi and Andreau, 2013). In this article, I selected decision-making as an example of executive function. Although neural mechanisms of decision-making have been examined using a variety of behavioral tasks including perceptual decision-making or value-based decision-making, free-choice decision-making is a typical example of a top-down control mechanism to which the prefrontal cortex contributes. In free-choice decision-making, the subject needs to select one option among others without any *a priori* information such as which option is better or worse. Since free-choice decision-making is performed without any prior knowledge of options, this decision-making must be a typical top-down operation and can be considered to be a fundamental process for the initial phase of any type of decision-making. Recent neuroimaging studies using human subjects have shown that the prefrontal cortex participates in free-choice decision-making. In this article, I will discuss the importance of free-choice decision-making for understanding the neural mechanisms of decision-making in general and how prefrontal neurons contribute to free-choice decision-making processes. A short-term active state has been observed just before presentation of the imperative stimulus in free-choice decision-making tasks (Marcos and Genovesio, 2016; Mochizuki and Funahashi, 2016). Since this short-term active state apparently biases the subject's subsequent decision, this is an important signal for free-choice decision-making. Therefore, I will discuss the cause of this short-term active state in prefrontal neurons and how this active state affects the subject's decision.

## Paradigms for examining neural mechanisms of decision-making

### Memory-based decision-making

Neural mechanisms for decision-making have been examined in a variety of experimental paradigms in animal studies. In one type of animal study, a particular sensory stimulus determines a particular behavioral response. For example, in delayed-response tasks (e.g., Funahashi et al., 1989) or delayed matching-to-sample tasks (e.g., Miller et al., 1996), the stimulus presented in the cue period determines the subsequent behavioral choice. The subject is required to remember the stimulus presented during the cue period in a given trial and to use this information to make a behavioral decision during the response period. Since the information that needs to be remembered during the delay period changes from trial to trial and since behavioral choice is always determined by the cue stimulus, the reward history and choice history associated with each cue stimulus have no value in this decision-making. The decision-making in these tasks only depends on the memory of the preceding cue stimulus. Therefore, this type of decision-making can be called *memory-based decision-making*.

### Value-based decision-making

Another type of decision-making is *value-based decision-making* (Rangel et al., 2008). The reward history and choice history associated with a particular option play important roles in this decision-making (e.g., Barraclough et al., 2004; Kennerley et al., 2006). When we need to select one option from among multiple known alternatives, we usually select an option that is associated with a higher reward value (i.e., a more satisfying, more pleasurable, or more valuable option). In this situation, the subject assigns each option a particular reward value based on learning and repeated experience. Therefore, each option is associated with a certain reward value, such that one option is assigned a higher reward value and another option is assigned a lower reward value. The assignment of a reward value to each option based on learning and repeated experience is included as part of the reward history and choice history of the option, and these histories could affect future selections. Learning mechanisms that establish a reward history can be explained using a reinforcement learning model. Each option is associated with an expected reward value. When the subject needs to select one option among multiple alternatives, the subject compares the expected values of the reward among the different options and makes a decision to choose the option having the highest expected value of the reward. The expected value associated with each option changes systematically, such that a positive outcome after the selection of a particular option increases its expected value, while a negative outcome decreases its expected value. This increase or decrease in the expected value is accumulated as the reward history of the option. If the choice of the option is associated with an increase or decrease in the expected value, the cumulative effect is called the choice history of the option. When the subject needs to make a decision to select one option from among others, the cumulative effect of the reward history for each option influences the subject's decision. For example, suppose that the subject tries to select either option A or B. If the selection of option A is expected to produce more satisfying and pleasurable results for the subject than the selection of option B (option A has a higher reward value than option B), option A would be selected more frequently than option B whenever the subject faces a selection between options A and B. Therefore, in value-based decision-making, the stimulus with a higher reward value would be selected more often by the subject. The reward history associated with each option plays an important role in value-based decision-making. Value-based decision-making is a typical form of decision-making that we perform in our daily life.

### Free-choice decision-making

**Figure 1:**
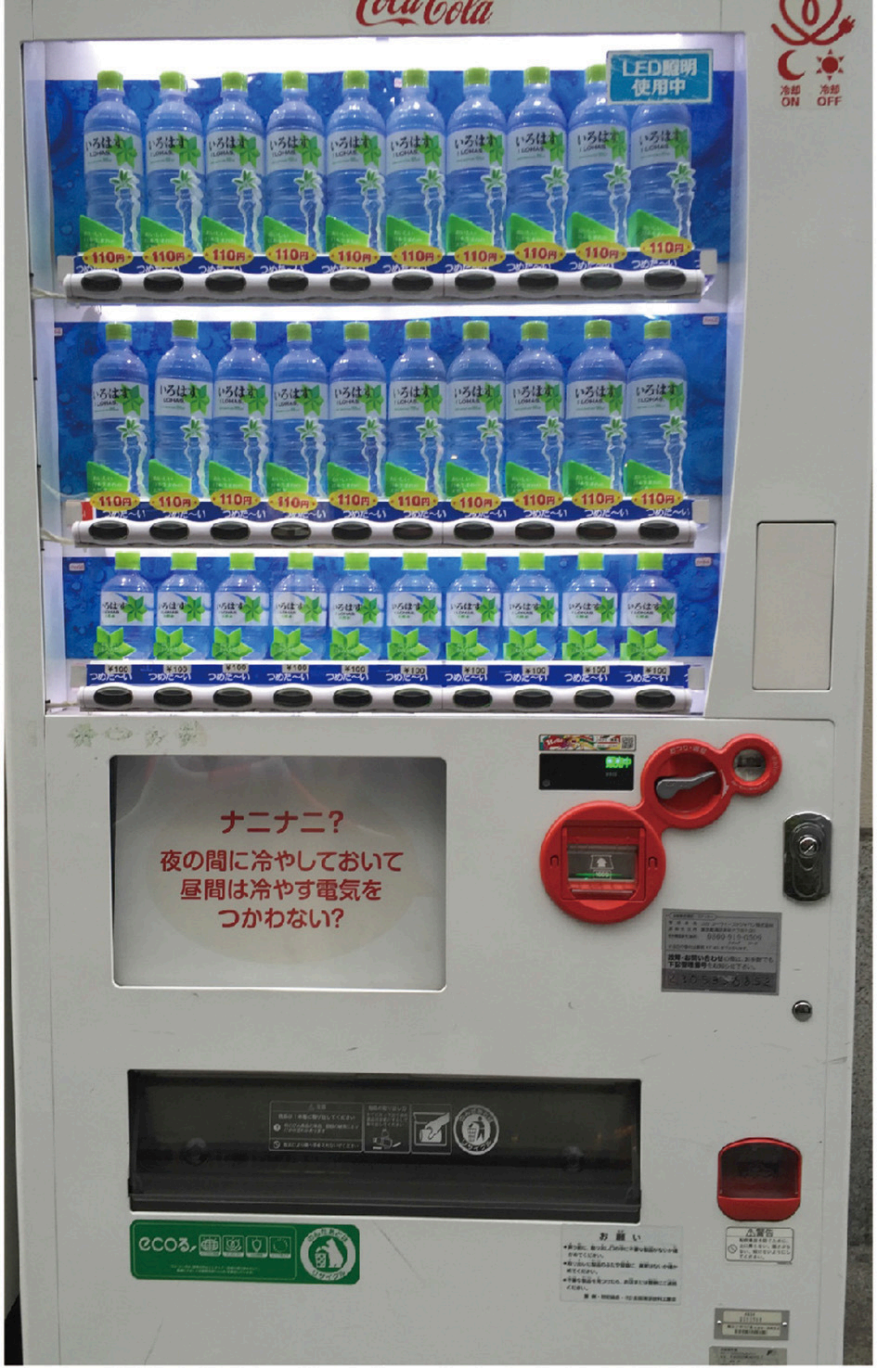
A vending machine in Japan.

Another type of decision-making is decision-making under free-choice conditions (*free-choice decision-making*). Suppose that you encounter a vending machine, as shown in Figure 1: which bottle will you choose? This particular vending machine dispenses bottles of mineral water, and all of the bottles are the same. These bottles have the same quality, the same quantity, and the same price. Therefore, whichever bottle you select, you will get the same outcome. In this decision-making, the reward history, choice history, and memory all have no effect on the decision. This is a typical situation in free-choice decision-making. In free-choice decision-making, the subject achieves the same outcome regardless of the option selected. Similarly, when we need to make a choice among unknown and untried options, we cannot make the decision based on the difference in the expected values among options. Therefore, we would perform free-choice decision-making in this situation.

### Importance of examining the neural mechanism of free-choice decision-making

When the subject performs a discrimination task, for example, value-based decision-making is used to select the option with a high reward value. In value-based decision-making, the accumulation of reward experience acquired by selecting a particular option plays an important role. However, at the very beginning of training of the discrimination task, the subject faces multiple unknown options. The subject has no information regarding which option is associated with the reward and thus has a higher reward value and is preferable. Even in this situation, the subject must select one option, and this selection is based on free-choice decision-making. However, after the subject makes a decision for several trials, they may eventually recognize which option has a higher (or lower) reward value, assign a certain reward value to each option, and tend to select the option associated with a higher reward value. Thus, although value-based decision-making plays an important role in the discrimination task, free-choice decision-making is always performed at the very initial phase of learning in the discrimination task. Whenever we face unknown and untried options and decide to select one, important factors in value-based decision-making, such as reward history, choice history, and memory of each option, do not provide any appropriate information regarding which option we should select. Thus, free-choice decision-making is a fundamental and prototypic form of decision-making and we always encounter the conditions that require free-choice decision-making at the initial phase of any kind of decision-making. Therefore, it is important to examine the neural mechanisms of free-choice decision-making. Such examination of the neural mechanisms of free-choice decision-making may provide valuable information for understanding the basic neural mechanisms of decision-making in general.

## Prefrontal contribution to free-choice decision-making: human imaging studies

Recent neuroimaging studies using human subjects have shown that the prefrontal cortex plays a significant role in spontaneous or self-generated behavior, and internally-driven decision-making (Frith et al., 1991; Hyder et al., 1997; Lau et al., 2004; Haynes et al., 2007; Soon et al., 2008). Spontaneous or self-generated actions are internally driven and not specified by external stimuli. Frith et al. (1991) used routine tasks, in which each response was specified by an external stimulus, and novel tasks, in which each response needed to be selected by the subject's willed action, and examined brain activation using PET (positron emission tomography). They found increased regional blood flow in the dorsolateral prefrontal cortex and the anterior cingulate cortex when subjects performed novel tasks in both a speaking-a-word condition and lifting-a-finger condition. Hyder et al. (1997) repeated the study done by Frith et al. (1991) using fMRI, and confirmed the bilateral activation of the dorsolateral prefrontal cortex in the willed action task (lifting-a-finger), although they observed only left dorsolateral prefrontal activation in the verbal task. Thus, although modality linked activation can be observed, the results obtained by Hyder et al. (1997) indicate that the dorsolateral prefrontal cortex plays a significant role in self-generated willed actions.

On the other hand, Haynes et al. (2007) used fMRI and examined whether the activity of the prefrontal cortex encodes a subject's current intention. In their task, human subjects were required to select either the addition or subtraction of two numbers by themselves and then provide the answer. In the task, when the cue word “select” was presented on the monitor, subjects were first required to choose either addition or subtraction of the numbers by themselves. After a variable delay (2.7–10.8 s), two numbers were presented on the monitor. Shortly after the presentation of two numbers, a response screen, which had four numbers (one was the answer of addition, one was the answer of subtraction, and the remaining two were incorrect numbers), was presented. While the response screen was presented, the subjects were required to select a correct number based on their choice of the calculation. The variable delay between the presentation of the cue word and the presentation of two numbers made the appearance of the two numbers unpredictable and forced the subject to maintain and prepare the chosen calculation during this period. Using this task, Haynes et al. (2007) obtained the spatial pattern of fMRI signals from the signals recorded from each local brain region, decoded information represented in these fMRI signals, and calculated decoding accuracies (how accurately MRI signals can predict the subject's decision regarding the calculation method) in many brain regions. As a result, during the delay period, they found high decoding accuracies in the anterior medial frontal cortex and the lateral prefrontal cortex. On the other hand, during the response period, high decoding accuracies were observed in the posterior medial frontal cortex (presumably the supplementary motor area). The lack of an explicit instruction suggesting which method the subjects had to select and the random arrangement of the four numbers on the response screen prevented the subjects from preparing behavioral responses during the delay period. Therefore, these results indicate that the activity of the prefrontal cortex reflects the subject's own mental state or intention and that this mental state or intention determines the subject's subsequent choice or action.

Soon et al. (2008) reported that the prefrontal cortex plays an important role in decision-making under free-choice conditions. They used a freely paced motor-decision task. In this task, the subjects were asked to freely decide to press one of two buttons by the left or right index finger. The subjects needed to gaze at the center of the screen, where alphabetical letters were presented one by one every 500 ms. At any moment, when the subject wanted to press either button, they could freely decide to press that button. At the same time, they were required to remember the letter that was presented on the screen when they decided to press the button. After they pressed either button, a response mapping screen, which had four letters, was presented. During the response period, they had to indicate when they made a decision by selecting the corresponding letter. Soon et al. (2008) examined the temporal change in decoding accuracy, which indicates how accurately information regarding which button would be pressed was decoded from local patterns of fMRI signals in various brain regions. They found that the frontopolar cortex (BA 10) and the medial parietal cortex (cortical area from the precuneus to the posterior cingulate cortex) exhibited high decoding accuracy before a conscious decision (when the selected letter had been presented on the screen), while the primary motor cortex and the supplementary motor area exhibited high decoding accuracy in the execution phase of the button press. Another study, in which the subjects needed to decide to press either the left or right button when cued by an external trigger, showed that the frontopolar cortex was the first cortical area where the actual decision was made. Thus, the results obtained by Soon et al. (2008) also indicate that the prefrontal cortex, especially the frontopolar cortex, participates in free-choice decision-making.

Lau et al. (2004) also showed that the dorsolateral prefrontal cortex contributed to decision-making in free-choice conditions. They compared the magnitude of activation among three choice conditions. In their FREE condition, human subjects were required to select one target randomly and move the cursor to the target. In their SPECIFIED condition, the subjects were required to move the cursor to the target with the same features as the cursor. In their ROUTINE condition, the subjects were required to move the cursor to the highlighted target. By comparing the fMRI signals in these three conditions, they concluded that the pre-supplementary motor area is closely associated with the free-choice of responses, because this area was activated only in the FREE condition. The dorsolateral prefrontal cortex was active in both the FREE and SPECIFIED conditions. The results obtained by Lau et al. (2004) indicate that the dorsolateral prefrontal cortex is associated with the free-choice of responses.

Although there have been few reported studies on the neural mechanisms of decision-making in free-choice conditions (Watanabe et al., 2006; Watanabe and Funahashi, 2007; Mochizuki and Funahashi, 2014, 2016; Marcos and Genovesio, 2016), these studies have indicated that the prefrontal cortex contributes to this decision-making. The supplementary motor area and the pre-supplementary motor area have been shown to participate in internally driven motor actions (Mushiake et al., 1991; Halsband et al., 1994; Cunnington et al., 2002; Nachev et al., 2008). The cingulate motor area (Shima and Tanji, 1998) and the dorsal anterior cingulate cortex (Walton et al., 2004) have also been shown to participate in behavioral responses based on the subject's own decision. As indicated by Soon et al. (2008), the prefrontal cortex, especially the frontopolar cortex and the dorsolateral prefrontal cortex, implicitly make a decision well before execution of an action. Therefore, the prefrontal cortex is thought to be a leading brain area for making spontaneous and self-generated behaviors and internally driven decision-making. Thus, the prefrontal cortex is thought to significantly contribute to decision-making in free-choice conditions, under which no external signal specifies a particular choice.

## Prefrontal contribution to free-choice decision-making: animal physiological studies

### Neural correlate of free-choice decision-making in the prefrontal cortex

Neuroimaging studies using human subjects have examined which brain areas participate in decision-making in free-choice conditions, and the results have indicated that the prefrontal cortex plays an important role in this type of decision-making. Neurophysiological studies using monkeys have also investigated the neural mechanism of free-choice decision-making in the prefrontal cortex (Watanabe et al., 2006; Watanabe and Funahashi, 2007; Mochizuki and Funahashi, 2014, 2016; Marcos and Genovesio, 2016) and cortical eye fields (Coe et al., 2002). Coe et al. (2002) used a free-choice delayed saccade task and examined single-neuron activity in the frontal eye field, the supplemental eye field and the lateral intraparietal cortex. In their task, while monkeys maintained fixation at a fixation point, two visual targets (one located within the receptive field and the other located outside it) were presented simultaneously. After the fixation point was turned off, monkeys were required to freely choose either target and make a saccade to that target. They found not only an enhanced response during visual target presentation but also stronger activation before visual target presentation (*anticipatory bias*) in all three eye fields when monkeys chose the target located within the receptive field. More neurons in the supplementary eye field exhibited stronger anticipatory bias and earlier activation than neurons in the frontal eye field and the lateral intraparietal cortex. Therefore, they concluded that the supplementary eye field plays more important roles in internally driven decision-making processes (Coe et al., 2002).

**Figure 2:**
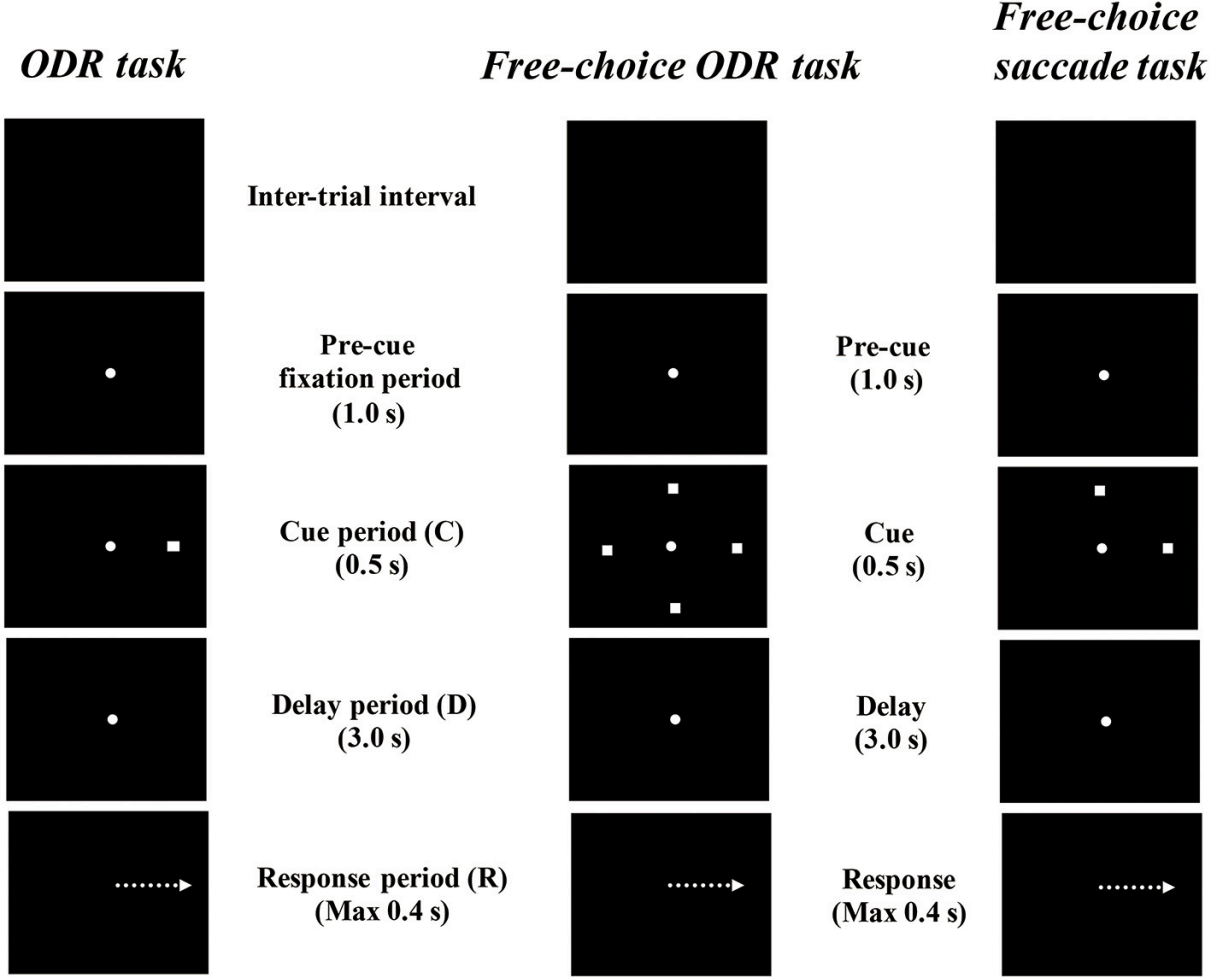
Diagrams of the temporal sequence of the behavioral tasks. An ordinary oculomotor delayed-response (ODR) task, a free-choice ODR task used by Watanabe et al. (2006) and Watanabe and Funahashi, and a free-choice memory-guided saccade task used by Mochizuki and Funahashi (2016).

**Figure 3:**
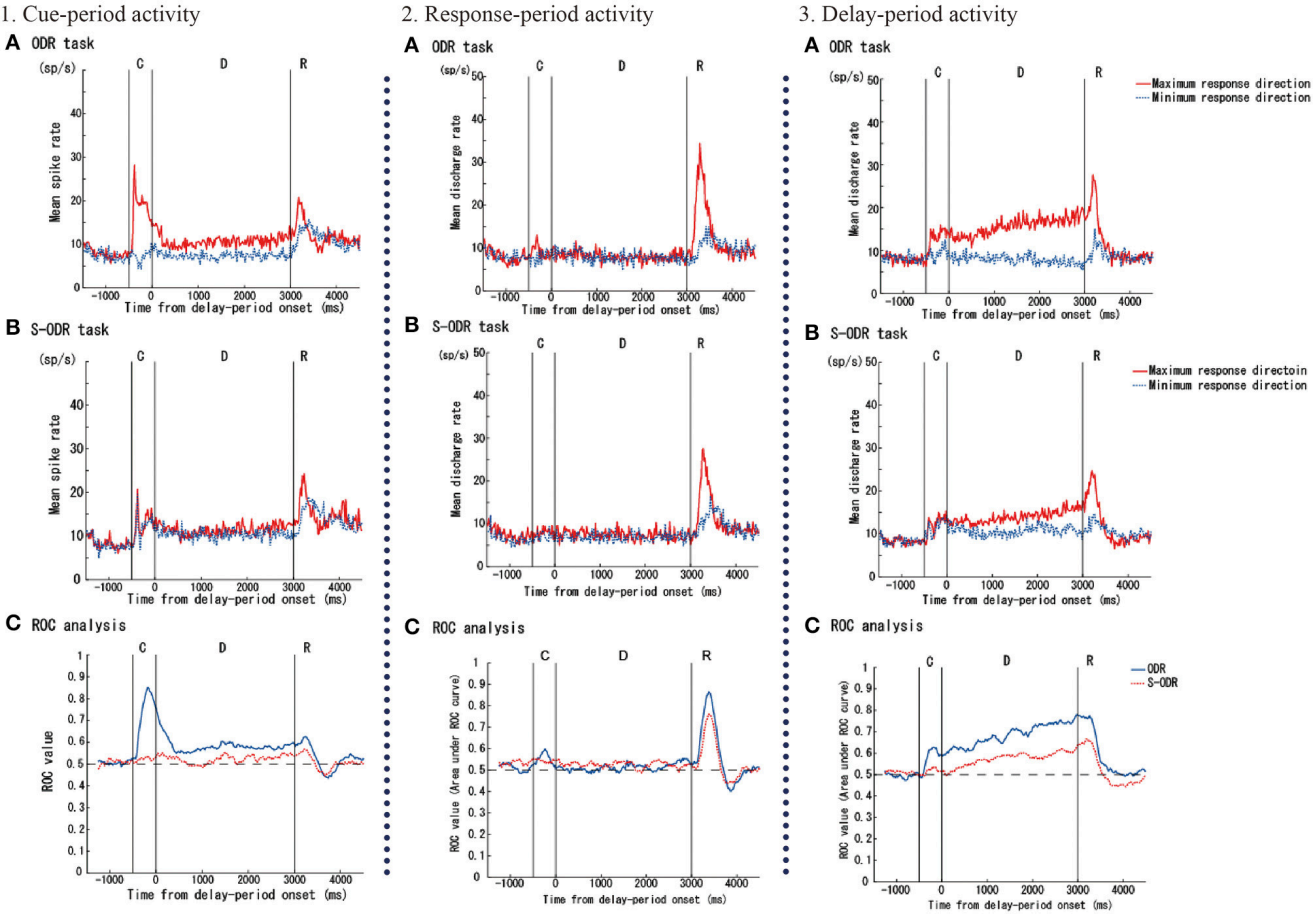
Examples of three kinds of task-related prefrontal activities (cue-, response-, and delay-period activities) observed while monkeys performed an ordinary oculomotor delayed-response (ODR) task (ODR task) and a free-choice ODR task (S-ODR task). In figures showing neural activities in the ODR task **(A)** and S-ODR task **(B)**, red lines indicate the activity when the visual cue was presented at the neuron's best (or maximum response) direction in the ODR task and when the monkey selected the neuron's best direction in the S-ODR task, while blue lines indicate the activity when the visual cue was presented at the neuron's worst (or minimum response) direction in the ODR task and when the monkey selected the neuron's worst direction in the S-ODR task. The bottom three figures **(C)** show the results of the ROC analysis comparing the differences in prefrontal activity between the best direction and the worst direction in the two task conditions (blue, ODR task; red, S-ODR task). C, D, and R indicate the cue-period, the delay period, and the response period, respectively. The lengths of the cue and delay periods were 500 and 3,000 ms, respectively. Figures are reproduced from Watanabe et al. (2006) with permission from the copyright holder.

Watanabe et al. (2006) examined prefrontal single-neuron activity while monkeys performed a free-choice saccade task (Figure 2). They asked monkeys to perform a modified version of an oculomotor delayed-response task (free-choice ODR task), in which monkeys were required to choose one of four identical visual cues and make a memory-guided saccade to the selected cue position after a 3-s delay period. The locations of the four visual cues were fixed during the experiment and these four visual cues were presented simultaneously during a 0.5-s cue period. Since monkeys received the same amount of the same reward regardless of whichever cue position they selected as a saccade target, they could freely choose any of the visual cues by themselves. First, they compared the monkeys' behavior between an ordinary oculomotor delayed-response task (ODR task) and a free-choice ODR task to determine when monkeys made a decision regarding which direction they make a saccade. In the ordinary ODR task, since the saccade direction is determined externally by the presentation of a visual cue during the cue period, monkeys can prepare their saccade direction in advance. Therefore, comparison of the saccade reaction times in the two tasks should reveal whether monkeys made the decision of the saccade direction before or after the presentation of the Go-signal in the free-choice ODR task. For example, if the monkey made this decision after the presentation of the Go-signal in the free-choice ODR task, the reaction times in this task would be longer than those in the ordinary ODR task. However, if the monkey made this decision well before the presentation of the Go-signal (e.g., during the cue period or the delay period) in the free-choice ODR task, the reaction times in this task should be similar to those in the ordinary ODR task. In fact, the reaction times in the free-choice ODR task were not significantly different from those in the ordinary ODR task, indicating that monkeys made the decision regarding the saccade direction before the response period (Watanabe et al., 2006). This was further supported by the observations that prefrontal neurons exhibiting saccade-related activity showed the same directional preference between these two tasks and that the temporal profiles of saccade-related activity were the same in these two tasks (Figure 3). Neurophysiological studies showed that neurons having directional cue-period activity in the ordinary ODR task did not show directional selectivity in the free-choice ODR task, suggesting that these neurons did not participate in decision-making regarding the saccade direction in the free-choice ODR task (Figure 3). However, prefrontal neurons having directional delay-period activity in the ordinary ODR task exhibited a similar directional preference in the free-choice ODR task (Figure 3). In addition, delay-period activity of these neurons gradually increased toward the end of the delay period in the free-choice ODR task (Watanabe and Funahashi, 2007). These results indicate that delay-period activity plays an important role in the decision for the saccade direction in the free-choice ODR task. Further, the gradual increase in delay-period activity toward the end of the delay period suggests the accumulation and integration of neural information for decision-making and that the decision for the saccade direction is made sometime during the delay period (Watanabe and Funahashi, 2007).

It has been shown that neurons having spatially selective delay-period activity participate in the animal's decision regarding the saccade direction (Coe et al., 2002; Watanabe et al., 2006; Watanabe and Funahashi, 2007). However, in these studies, a fixed set of spatial targets were repeatedly presented as saccade targets in a block of trials. In this situation, monkeys generally tended to choose the same target repeatedly for several consecutive trials, because the monkey received the same reward regardless of the target it chose. In the study by Coe et al. (2002), two visual targets were presented at the same locations throughout the entire recording period of a given neuron. To prevent monkeys from showing this tendency, the researchers introduced specific reward schedules, such that, when the monkey selected the same target repeatedly, the amount of reward first increased, peaked, and then began to decrease. As a result, the monkeys frequently chose the same target for several consecutive trials until the amount of reward peaked. Therefore, they considered that anticipatory bias could represent neural processes of voluntary attention or motor preparation, such that the monkeys had already decided the saccade direction and allocated attention to a specific target by the time that the visual targets appeared (Coe et al., 2002). Similarly, Watanabe et al. (2006) and Watanabe and Funahashi (2007) set a maximum repetition frequency for the same target selection to prevent the monkeys from showing this general behavioral tendency. If the monkeys made a saccade to the same target repetitively for four consecutive trials, they would not receive a reward if they chose the same target again. Although this procedure forced the monkeys to choose another target, the monkeys already decided upon the saccade direction by the time that the visual targets appeared during repetitive selection of the same target. Therefore, these experimental situations were not ideal for examining the neural mechanisms of decision-making in free-choice conditions.

### Choice-predictive activity in the prefrontal cortex

To improve the experimental conditions and establish free-choice conditions behaviorally, Mochizuki and Funahashi (2014, 2016) established a free-choice memory-guided saccade task in which the monkeys were required to choose either of two targets for a memory-guided saccade by themselves (Figure 2). In every trial of this task, the locations of two saccade targets were randomly selected from among multiple predetermined locations by a computer. Since the pair of locations assigned as saccade targets changed from trial to trial, monkeys could not predict which pair would be presented in a given trial. Although the monkeys obtained the same reward regardless of the location they chose, this procedure almost entirely eliminated the monkey's behavioral tendency to choose the same target repeatedly (Mochizuki and Funahashi, 2014, 2016).

**Figure 4:**
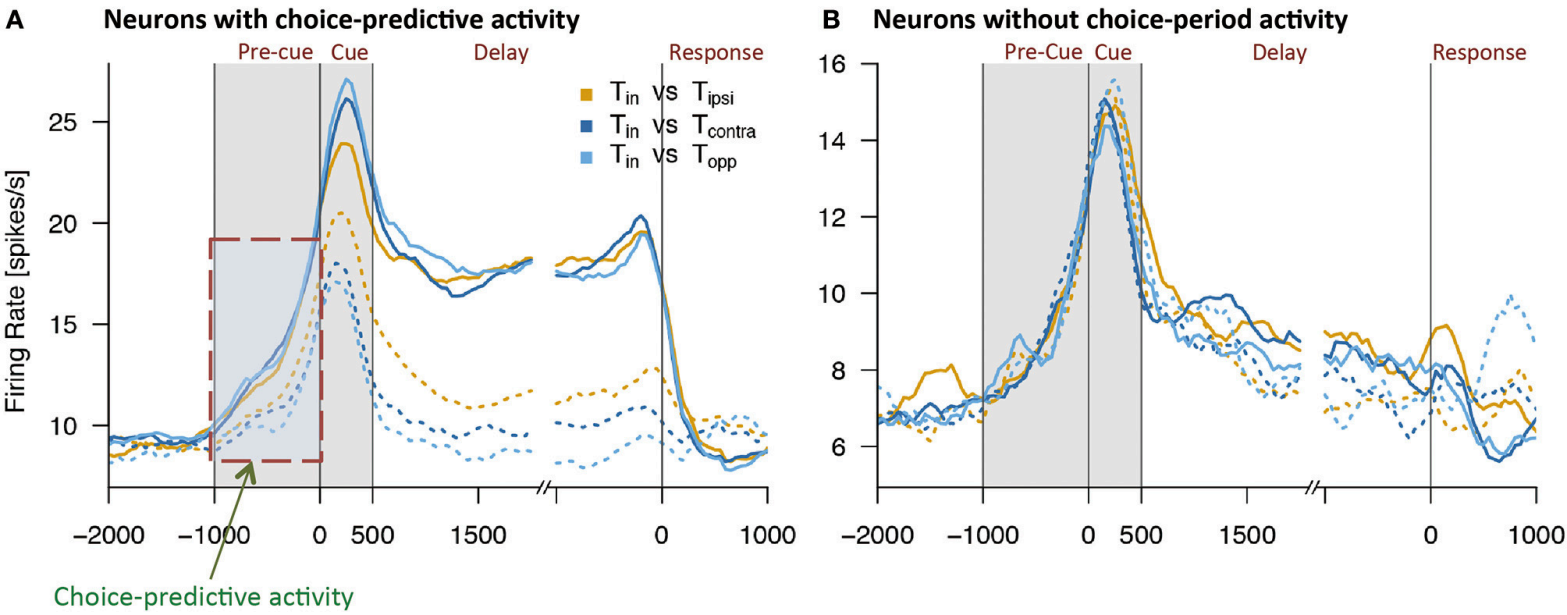
Examples of activities observed in two groups of prefrontal neurons while monkeys performed free-choice tasks. **(A)** Temporal patterns of activities observed in prefrontal neurons with choice-predictive activity. This neuron exhibited significant choice-predictive activity and delay-period activity when the monkey selected saccade directions toward the neuron's preferred direction (solid lines), regardless of wherever the remaining cue was presented (dotted lines). **(B)** Temporal patterns of activities observed in prefrontal neurons without choice-predictive activity. This neuron exhibited the same temporal patterns of activity regardless of whether the monkey selected saccade directions toward the neuron's preferred direction (solid lines) or non-preferred directions (dotted lines). T*in* indicates the monkey's selection of the neuron's preferred direction (solid lines) and T*ipsi*, T*contra*, and T*opp* indicate the monkey's selection of the neuron's non-preferred directions (dotted lines). Figures are reproduced from Mochizuki and Funahashi (2016) with permission from the copyright holder.

Using this method, Mochizuki and Funahashi (2016) examined neural mechanisms related to decision-making in free-choice conditions in the prefrontal cortex. They found that a visual response was significantly enhanced when the monkey eventually chose the saccade target presented within the neuron's receptive field (Figure 4). This enhancement was not observed when the monkey chose the saccade target presented outside of the receptive field, even though the other visual target was presented within the receptive field simultaneously. Interestingly, the enhancement of neural activity began several 100 ms before presentation of the visual target only in trials in which the monkey eventually chose the visual target presented within the neuron's receptive field. Since this enhanced activation occurred before cue presentation and since this activation occurred or not predicted the animal's decision regarding the subsequent saccade direction, this activity was called *choice-predictive activity* (Mochizuki and Funahashi, 2016). Coe et al. (2002) reported anticipatory bias, which has features similar to those of choice-predictive activity. However, choice-predictive activity is different from anticipatory bias, since no information regarding cue locations was provided during the pre-cue fixation period and, in any given trial, two cue locations were selected randomly from among multiple locations. In addition, the reward was always the same regardless of the target the monkey selected across trials. Therefore, choice-predictive activity may not be a result of voluntary attention or motor preparation for a specific response, but rather could represent an active state of prefrontal neurons immediately before cue presentation.

Activity similar to choice-predictive activity was reported by Marcos and Genovesio (2016). They used a strategy task, in which the monkey needed to choose either a repeat-stay (make a saccade to the same target selected in the preceding trial) or a change-shift (make a saccade to a different target from the one selected in the preceding trial) strategy depending on whether or not the same imperative stimulus was presented repeatedly. In the change-shift strategy, the monkey was required to select one of two targets without knowing which target was associated with a reward. Therefore, they considered selection of the change-shift strategy to be the free-choice condition. They observed a significantly different magnitude of activity ~200 ms before presentation of the stimulus depending on whether or not the monkey eventually selected the target in the neuron's receptive field. They called this activity *prestimulus activity*. Since prestimulus activity seems to have characteristics similar to those of choice-predictive activity, these activities may be caused by the same mechanism.

### What causes choice-predictive activity?

Choice-predictive activity might reflect a transient active state caused by the spontaneous fluctuation of baseline activity observed in prefrontal neurons. As stated before, during the pre-cue fixation period, the subject only looked at the fixation target without any other external stimulus, and no information regarding the direction of the impending response was provided. Therefore, choice-predictive activity is not associated with voluntary attention to a particular stimulus or motor preparation for a particular response. Most prefrontal neurons exhibit 1–10 spikes/s of irregular and arrhythmic spontaneous activity (Fuster, 1973; Quintana et al., 1988). This irregular spontaneous change in baseline activity could temporarily alter the neuron's active state. Therefore, choice-predictive activity could be explained as the result of a short-term active state caused by spontaneous irregular fluctuation of the neuron's baseline activity. The unexpected transient increase in activity caused by spontaneous fluctuation of the neuron's baseline activity could add to the normal response to the visual cue presentation and produce a stronger visual response than usual. This stronger response to the visual cue could produce a bias in subsequent information processes, especially for determining the direction of the response.

A similar biasing effect via the spontaneous fluctuation of baseline activity on the subsequent choice has been reported (Platt and Glimcher, 1999; Shadlen and Newsome, 2001). For example, Shadlen and Newsome (2001) examined the activities of lateral intraparietal (LIP) neurons while monkeys performed a perceptual decision-making task using random-dot motion, and showed that, when it was difficult for the monkey to discriminate the direction of dot motion because of very low motion coherence, these neurons often exhibited higher discharge rates just before the onset of random-dot motion stimulus in trials in which the monkey eventually chose the neuron's preferred motion direction. They suggested that this higher discharge rate just before the onset of the motion stimulus is caused by the spontaneous fluctuation of baseline activity, and this activation affected and biased the subsequent competition among LIP neurons, each of which represented a different direction of visual motion. By a theoretical approach using an integrate-and-fire neuron network model, Rolls and Deco (2011) showed that a biasing effect can be produced by fluctuation of the noise inputs to the network and this biasing effect actually affects the output of the network. Their study confirmed that a small change in the activation level in the network produced by the fluctuation of spontaneous neural activity affects the competition among networks, each of which represents different information processing, and biases network selection.

Thus, choice-predictive activity observed by Mochizuki and Funahashi (2016) could be a transient active state caused by the irregular fluctuation of spontaneous neuron activities. Pre-stimulus activity reported by Marcos and Genovesio (2016) could also be explained by the same mechanism. A transient increase in spontaneous discharge occurred in neurons exhibiting a directional visual response just before presentation of the visual cue. When two visual cues (one in the neuron's receptive field and the other outside of it) are presented simultaneously, this transient increase could be added to the normal visual response to the visual cue presented in the neuron's receptive field and this increase would enhance the visual response and produce a larger response than usual. As a result, this enhanced visual response would produce a bias in the selectivity toward the neuron's best direction, and eventually affect the direction of the behavioral response in decision-making.

### Functional relations between choice-predictive activity and delay-period activity

Delay-period activity is known to play an important role in performance of the ODR task (Funahashi et al., 1989, 1993; Funahashi, 2015). Prefrontal neurons exhibiting significant choice-predictive activity in the free-choice task exhibited directional delay-period activity in the ODR task. However, neurons that did not exhibit choice-predictive activity did not exhibit either delay-period activity itself or directional selectivity in delay-period activity in the ODR task (Mochizuki and Funahashi, 2016) (Figure 4). In addition, prefrontal neurons with firing that tended to be sustained during the fixation period in the ODR task tended to exhibit choice-predictive activity in the free-choice ODR task (Mochizuki and Funahashi, 2016). These results suggest that the firing properties of prefrontal neurons contribute to decision-making in the free-choice ODR task. Since delay-period activity is tonic sustained activity observed during the delay period, neurons exhibiting delay-period activity may have some specific intrinsic mechanism to support a sustained active state, such as local cortical circuits operating through a balance of excitation and inhibition generated by local recurrent connections (Shu et al., 2003). The operation of these circuits has been shown to generate self-sustained activity that is turned on and off by synaptic inputs (Shu et al., 2003). This intrinsic mechanism might help to maintain the active state caused by a transient increase in spontaneous discharge for a longer time (e.g., 100–200 ms). If this short-term maintenance of the active state occurred just before visual cue presentation, this active state might enhance the subsequent response to the visual cue presented in the neuron's receptive field. Since prefrontal neurons having choice-predictive activity exhibited directional delay-period activity, the enhanced visual response might trigger the generation of directional delay-period activity. Thus, basic neural properties that contribute to working memory mechanisms could also contribute to the neural mechanisms for decision-making in the free-choice ODR task.

## Neural mechanism for free-choice decision-making in the prefrontal cortex

**Figure 5:**
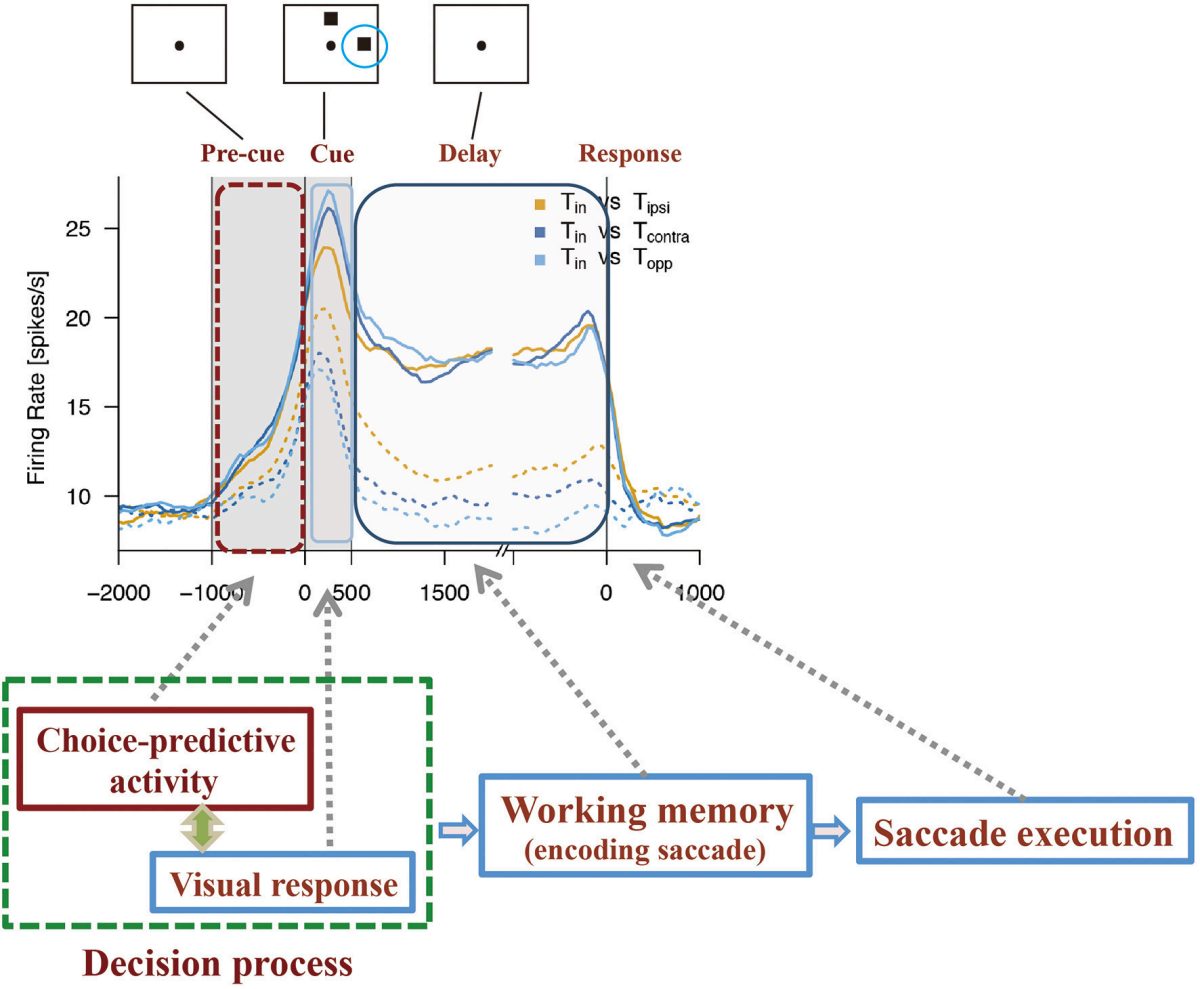
Diagram to explain how choice-predictive activity could contribute to decision-making in free-choice conditions. Figures are reproduced from Mochizuki and Funahashi (2016) with permission from the copyright holder.

Based on the observation of choice-predictive activity (Mochizuki and Funahashi, 2016) and prestimulus activity (Marcos and Genovesio, 2016) and their possible causes, the mechanism shown in Figure 5 can be considered as a prefrontal neural mechanism for free-choice decision-making. As discussed before, the firing properties of prefrontal neurons have distinctive features. First, every prefrontal neuron exhibits irregularly fluctuating spontaneous firing. This irregular fluctuation of spontaneous firing can occur at any time during the task in any given trial. Second, some prefrontal neurons have an intrinsic mechanism that produces a specific firing property. These neurons can maintain an activated state probably for several 100 ms after one or few spikes occur. Therefore, these neurons tend to exhibit a tonic sustained firing pattern with a transient increase in the discharge rate. This property is an important feature of prefrontal neurons that exhibit tonic sustained delay-period activity.

While monkeys perform ODR tasks, most prefrontal neurons exhibit directionally selective activities during the cue, delay, and response periods (Funahashi et al., 1989, 1990, 1991; Funahashi, 2014, 2015). During the pre-cue fixation period, a monkey only looks at the central fixation target, and any other external stimulus that might cue the monkey to prepare a behavioral response is not presented. Therefore, the condition of each prefrontal neuron is thought to be stable during the pre-cue fixation period across trials. However, since every prefrontal neuron exhibits irregularly fluctuating spontaneous firing, an unexpected transient increase in firing can occur any time during the pre-cue fixation period. If such a transient increase in firing happens to occur just before visual cue presentation in neurons having directional cue-period activity, this active state would be maintained for a moment until the early phase of the cue period and could enhance the visual response to the visual cue. Since each visual neuron has a distinct spatial representation (e.g., visual receptive field or directional selectivity), such enhancement of the visual response could strengthen and enhance the neuron's spatial representation. When two visual cues are presented simultaneously in the free-choice ODR task and if one of these is presented within the neuron's receptive field, since this neuron is in a more active state, this neuron would be able to respond more quickly and more strongly to the visual cue presented in its receptive field. It has been shown that neurons exhibiting different spatial selectivities have mutual inhibitory connections between them (Rao et al., 1999; Wang et al., 2004). Therefore, it can be considered that competitive interaction must be present among these neurons to make a final output. Inhibitory interactions are present among neurons and a neuron that exhibits faster and stronger activation in response to the stimulus is the winner in a winner-take-all competition (Compte et al., 2000; Wang, 2008). Such a winner-take-all competition probably occurs among neurons having directional cue-period activity. As a result of this competition, one group of prefrontal neurons that exhibit a particular directional selectivity become winners and the directional information represented by these neurons is the final direction of the forthcoming behavioral response.

Thus, choice-predictive activity could be a transient activation produced by the irregularly fluctuating spontaneous discharge of prefrontal neurons. This activity causes a bias in the strength of the spatial representation of neurons. This bias affects the decision regarding the final output. Therefore, choice-predictive activity or prestimulus activity should be an important neural activity in free-choice decision-making. Thus, transient activation that occurs in certain neurons just before cue stimulus presentation and a winner-take-all competition among neurons, each of which has a different stimulus selectivity, are the two main components for understanding the neural mechanisms of free-choice decision-making.

## Prefrontal contribution to other types of decision-making

Neural mechanisms related to various types of decision-making have been examined in the prefrontal cortex. In experiments using monkeys, several behavioral paradigms including perceptual decision-making, value-based decision-making, decision-making under competitive conditions, and decision-making in conflict conditions have been used to examine prefrontal involvement in decision-making.

### Neural mechanisms of perceptual decision-making in the prefrontal cortex

In the perceptual decision-making paradigm, subjects are asked to report a direction of motion by using their eye movements when they see randomly moving dots with different levels of motion coherence (e.g., Shadlen and Newsome, 2001), or they need to identify whether a picture shows a dog or cat when they see a picture of a dog or cat that has been distorted to various degrees (e.g., Freedman et al., 2001, 2002), or they must state whether or not the current stimulus frequency is the same as that of the preceding stimulus frequency (e.g., Romo et al., 2002; Romo and Salinas, 2003). In this paradigm, a stimulus with various degrees of ambiguity is presented to the subject and the subject is asked to identify the stimulus itself or a feature of the stimulus. In the most difficult condition of this paradigm, a stimulus with the highest degree of ambiguity is presented to the subject, and it is impossible for the subject to identify the stimulus or its features. Therefore, decision-making in this condition is similar to decision-making in free-choice conditions.

The perceptual decision-making paradigm has been widely used to examine neural mechanisms related to decision-making. Especially in the lateral intraparietal cortex (areas LIP), single-neuron studies have been performed while monkeys performed motion discrimination tasks with a saccade response under various conditions (Shadlen and Newsome, 2001; Mazurek et al., 2003; Huk and Shadlen, 2005; Hanks et al., 2006; Churchland et al., 2008; Kiani and Shadlen, 2009; Shadlen and Kiani, 2013). The neural mechanisms of perceptual decision-making in a motion discrimination task have also been examined in human subjects by fMRI (Kayser et al., 2010). Theoretical considerations based on the results obtained from single-neuron studies (Gold and Shadlen, 2007; Furman and Wang, 2008; Wang, 2008) and a comparison of perceptual decision-making among rodents, monkeys, and humans (Hanks and Summerfield, 2017) have also been presented.

The neural mechanisms of perceptual decision-making have also been examined in the monkey prefrontal cortex using a motion discrimination task with a saccade response. For example, Kim and Shadlen (1999) used a perceptual decision task in a two-alternative forced-choice motion discrimination paradigm. In this task, the monkeys were required to discriminate the directions of random dot motions presented on a monitor and report the perceived direction using a saccadic eye movement to one of two visual targets. The random dots appeared outside the neuron's receptive field. Motion direction was either toward or away from the receptive field. Motion strength was varied systematically by changing the coherence of motion direction of the dots. The magnitude of the activity was affected by the motion strength, the motion direction, and the direction of the impending saccade in many prefrontal neurons. Kim and Shadlen (1999) calculated an index of predictive activity, which estimated the probability that an ideal observer could predict the monkey's decision based on the discharge rate, and showed that most neurons (86%) reliably predicted the monkey's choice during the motion-viewing period or the subsequent delay period. In addition, Kim and Shadlen (1999) showed that the activity predicted the monkey's decision 100–200 ms after the onset of random dot motion. Further, this predictive activity was observed even in the most difficult condition (no coherent motion condition) and its magnitude increased as motion coherence increased (easier condition) (Kim and Shadlen, 1999). Thus, these results indicate that prefrontal neurons contribute to the decision regarding the saccade direction in the two-choice perceptual decision-making task.

Ding and Gold (2012) examined single-neuron activity in the frontal eye field while monkeys performed perceptual decision-making using a motion discrimination task with a saccade response, and observed predictive activity with features similar to those of prefrontal activity. Predictive activity has also been observed in the parietal cortex (area LIP) while monkeys performed the same behavioral task (Shadlen and Newsome, 2001). In area LIP, predictive activity began early in the motion-viewing period. This activity was observed even in the most difficult condition and the timing and magnitude of the activity were affected by the strength of motion coherence (Shadlen and Newsome, 2001). These results indicate that comparison of the outputs of different populations of sensory neurons with a specific stimulus selectivity could be a mechanism for perceptual decision-making in higher-level brain regions, such as the prefrontal cortex and the parietal cortex (Kim and Shadlen, 1999; Gold and Shadlen, 2000; Shadlen and Newsome, 2001). In these perceptual decision-making tasks, a decision can be made in prefrontal and LIP neurons by comparing the difference in output activities between two groups of MT neurons: those sensitive to one direction of motion and those sensitive to another direction of motion. In addition to the comparison of output activities between the two groups of neurons, the integration or accumulation of output activity is also considered to be an important component of decision-making (Gold and Shadlen, 2000, 2007; Mazurek et al., 2003; Huk and Shadlen, 2005). Shadlen and Kiani (2013) used bounded evidence accumulation to explain neural mechanisms of perceptual decision-making in the LIP. The difference in the firing rate of MT neurons is proportional to motion strength, because the actual firing rate of MT neurons can be assessed using an average of spontaneous firing rates and the firing rate proportional to the strength of visual motion toward their preferred direction. LIP neurons, which receive the outputs of MT neurons, exhibit dynamic changes (decision-related changes) in the firing 100–200 ms after the onset of random dot viewing. This suggests that the firing rate of LIP neurons approximates the accumulation of the difference between two groups of MT neurons, each of which exhibits a preference for a different direction of motion. When this difference is accumulated until it reaches a certain boundary, it leads to a certain decision.

The predictive activity observed even in the most difficult condition reflected the monkey's behavioral response (saccade direction). In addition, this activity predicted the decision 100–200 ms after the presentation of dot motion in both prefrontal and LIP neurons. The most difficult condition in the perceptual decision-making paradigm is considered to be similar to the condition in free-choice decision-making. Therefore, this predictive activity is thought to represent the neural mechanism of free-choice decision-making and should correspond to a transient activation caused by spontaneous fluctuation of the baseline discharge rate.

### Neural mechanisms of value-based decision-making in the prefrontal cortex

The other paradigm used in animal studies is value-based decision-making. Decision-making is an evaluation process for making a particular choice from among a set of alternatives. A particular choice usually predicts a particular outcome or value. The outcome that results from a choice can sometimes be beneficial and rewarding for the subject, or sometimes risky and costly. Therefore, decision-making includes neural processes for evaluating risks and rewards, or costs and benefits. The link between a particular choice and a particular outcome can be achieved by learning based on the subject's experience or history of a particular choice and the outcome associated with this choice. This process can be described using the reinforcement learning model (Lee et al., 2012). Therefore, the subject's decision in a given situation can be made by assessing the relative value of each option. As a result of this process, the subject would select the option that could be expected to provide the most value.

For example, the monkey was asked to perform a two-choice visually-guided saccade task. In this task, both targets were presented during the response period and the monkey was required to freely select either target to get a reward. However, selection of one particular target resulted in reward delivery at a high probability, while selection of the other target resulted in either no reward delivery or reward delivery at only a low probability. Under this reward schedule, the monkey apparently evaluated the value of each target based on the choice history and continued to select the target with a high value (Platt and Glimcher, 1999; Dorris and Glimcher, 2004; Sugrue et al., 2004, 2005). These studies also showed that the monkey's target of choice changed systematically depending on the magnitude of reward probability assigned to each target, such that the monkey tended to choose the target associated with a higher reward value and maintained this selection until the reward contingency changed. Thus, these behavioral observations support the notion that monkeys use a value-based decision-making strategy in this task.

The activity of parietal neurons has been analyzed using this task to understand the neural mechanisms of value-based decision-making. The activity of parietal neurons is sensitive to the reward probability associated with a particular response. The subject's choice and the magnitude of the activation of parietal neurons are correlated with the relative amount of expected outcome associated with each response (Platt and Glimcher, 1999; Sugrue et al., 2004). On the other hand, Dorris and Glimcher (2004) indicated that the activity of parietal neurons was correlated with the subject's relative subjective desirability of a particular action, regardless of the specific combination of reward magnitude, reward probability, and response probability associated with each action.

The prefrontal contribution to value-based decision-making has been extensively examined in human neuroimaging studies (Krawczyk, 2002; Walton et al., 2004; Kennerley et al., 2006; Glascher et al., 2009; Rushworth et al., 2011). These studies indicate that several regions of the prefrontal cortex play distinct and important roles in reward-based decision-making: the ventromedial prefrontal cortex, the medial orbitofrontal cortex, the lateral orbitofrontal cortex, the anterior cingulate cortex, and the lateral anterior prefrontal cortex (Rushworth et al., 2011). Animal studies also show prefrontal participation in value-based decision-makings. Kennerley et al. (2011) indicated that the prefrontal cortex participates in value computation since many prefrontal neurons represented chosen value. However, they also showed that the anterior cingulate cortex and the orbitofrontal cortex encode different types of chosen value, such that the orbitofrontal cortex evaluates current choices whereas the anterior cingulate cortex encodes choice prediction and prediction error. Similarly, Rich and Wallis (2016) showed that the orbitofrontal cortex computes a value for each option. Further, Rudebeck et al. (2013) showed that the orbitofrontal cortex has a role to update a value of the option. Since the orbitofrontal cortex has been known to participate in specialized roles of reward-guided behaviors (Kringelbach, 2005), these results well agree with the notion that the orbitofrontal cortex participates in value-based decision-makings (Wallis, 2012). In addition, Donahue and Lee (2015) indicated that the dorsolateral prefrontal cortex also contributes to value-based decision-making, such that task-related activities were affected by reward outcomes so as to favor the choices that maximize reward. Thus, although the prefrontal cortex participates in value-based decision-making, contributions to this decision-making seem to be different in each sector of the prefrontal cortex.

### Decision-making under competitive conditions

As described in the previous section, decision-making is a process for making a particular choice from a set of alternatives. If a particular choice always predicts a particular outcome or value, the decision to select the option with the best outcome is optimal. Therefore, the best decision in a particular environment will depend on the subject's own choice history or reward history. However, in a natural environment, we cannot always make a decision based only on our own choice history or reward history. In the presence of competition, our decisions are often affected by the decisions of others.

A game is a typical situation for this type of decision-making. A game is usually played by multiple players and a payoff table specifies the amount of reward or penalty for each player based on the decisions made by all players. To win a competitive game, each player needs to find an optimal strategy by constantly changing the choice strategy. However, Nash (1950) showed that a competitive game with multiple players has at least one equilibrium condition in which no players can obtain any benefit by changing their choice strategies individually. This equilibrium condition is called the Nash equilibrium (Nash, 1950). Barraclough et al. (2004) asked two rhesus monkeys to perform an oculomotor free-choice task and play a game analogous to matching pennies against a computer. In their task, monkeys needed to select either the right or left visual target presented on the monitor by a saccadic eye movement. The monkeys were rewarded when they selected the same target as that selected by the computer. Three task conditions were used. In condition 0, the computer selected the target randomly with an equal probability (the reward rate was 0.5) regardless of the monkey's choice patterns. Since the reward rate was fixed at 0.5, both monkeys exhibited a spatial bias in their target selection (e.g., more frequent selection of the right target). In condition 1, the computer analyzed the monkey's choice history, not the reward history, while, in condition 2, the computer analyzed both the monkey's choice and reward histories. In these two conditions, the monkey's selection of the right target was closer to 0.5, which agreed with the Nash equilibrium in the matching penny game (Barraclough et al., 2004). The probability of the target choice was not affected by the choice in the preceding trial. However, the monkey's choice was influenced by the computer's choice in the preceding trial. Barraclough et al. (2004) applied a reinforcement learning algorithm to explain their behavioral results obtained in two monkeys and concluded that the monkeys approximated the optimal decision strategy using a reinforcement learning algorithm.

Barraclough et al. (2004) then examined dorsolateral prefrontal single-neuron activities while monkeys performed this free-choice task. As supporting behavioral observations, the activity during the fore-period and sometimes during the delay period was affected by the monkey's choice in the preceding trial. In addition, the signals related to the combination of the monkey's previous choice and its outcome were processed differentially in prefrontal neurons depending on the type of decision-making by the monkey. Since the monkeys flexibly changed their strategy based on the choice and reward histories and the opponent's choice strategy, these results support the notion that the prefrontal cortex plays a key role in optimizing the strategy for decision-making. Further, Seo et al. (2007) examined prefrontal activities while monkeys performed a similar penny-matching game and found neural signals that would influence the monkeys' forthcoming choice behavior. Thus, the results obtained by Barraclough et al. (2004) and Seo et al. (2007) indicate that the prefrontal cortex plays an important role in neural mechanisms of decision-making and that neural signals that affect forthcoming decision-making play a role in optimizing the strategy of decision-making under competitive conditions. However, in these studies, monkeys played against the computer. Therefore, the monkeys might not fully understand that the game was competitive. Recently, prefrontal single-neuron activities have been examined in social and real competitive conditions of two monkeys (Yoshida et al., 2012; Chang et al., 2013; Haroush and Williams, 2015). These studies indicated that different sectors of the prefrontal cortex participate in different functions in decision-making under social and competitive conditions. Further studies may need to understand prefrontal contribution to decision-making under competitive conditions.

### Decision-making in conflict conditions

When a subject is exposed to a high-conflict condition immediately preceding a trial, the task-relevant information is enhanced while the task-irrelevant information is suppressed. Therefore, the detrimental effect on performance produced by the conflict is reduced. This reduction in conflict is often called behavioral adaptation and has an advantageous effect in decision-making. Mansouri et al. (2007) showed that non-human primates with dorsolateral prefrontal lesions exhibited impaired behavioral adaptation when they performed a Wisconsin Card Sorting Test (WCST) analog. Mansouri et al. (2015) also showed that the dorsolateral prefrontal cortex holds information regarding the occurrence of the conflict in working memory and indicated that this information is used for optimal decision-making in a dynamic environment. Similarly, Boschin et al. (2017) applied repeated Transcranial Magnetic Stimulation (rTMS) to the dorsolateral prefrontal cortex to interfere with its activity while human subjects performed a conflict version of the WCST analog, and showed that the dorsolateral prefrontal cortex is a fundamental structure for optimal conflict-induced behavioral adaptation that maintains conflict-history information online across trials.

## Conclusions

Decision-making is an important executive function that involves the prefrontal cortex. Although the prefrontal cortex participates in various types of decision-making, an understanding of the neural mechanisms related to free-choice decision-making is important for understanding the basic neural mechanisms for various types of decision-making because free-choice decision-making is very basic and is part of the very first phase of other types of decision-making. Recent neuroimaging studies have shown that the prefrontal cortex plays an essential role in free-choice decision-making. Neurophysiological studies using monkeys performing decision-making tasks under free-choice conditions showed that, when the monkey was asked to make a decision to choose one option among other alternatives on its own, prefrontal neurons showing selectivity to that option exhibited transient activation just before presentation of the imperative cue indicating that option. This transient activation during the pre-cue fixation period has been called choice-predictive activity (Mochizuki and Funahashi, 2016) or prestimulus activity (Marcos and Genovesio, 2016). Studies suggest that this transient increase in activity is caused by the irregular fluctuation of spontaneous firing just before cue presentation, and that this increase in activity enhances the response to the cue presentation and biases the strength of the neuron's selectivity to the option. In addition, this biasing effect was observed only in neurons exhibiting sustained delay-period activity, and these prefrontal neurons are considered to have some specific intrinsic firing properties; i.e., these neurons tend to maintain an active state for a slightly longer time after a spike discharge. These results indicate that the biasing effect caused by transient activation is an important neural component of free-choice decision-making and that this biasing effect not only influences the animal's decision regarding the upcoming choice but is also linked with working memory mechanisms in the prefrontal cortex. Recently, Katz et al. (2016) indicated that decision-related activity observed in LIP neurons is not essential in the final outcome of perceptual decision-making and may reflect some secondary processes. Therefore, further studies may need to confirm that the biasing effect by transient activation in prefrontal neurons having directional activity is causally related to free-choice decision-making.

## Author contributions

The author confirms being the sole contributor of this work and approved it for publication.

### Conflict of interest statement

The author declares that the research was conducted in the absence of any commercial or financial relationships that could be construed as a potential conflict of interest.
